# Metabolomic Profiling Reveals Geographical Origin, Tissue-Specific Specialization, and Environmental Plasticity in Secondary Metabolism of *Dendrobium officinale*

**DOI:** 10.3390/metabo16040279

**Published:** 2026-04-20

**Authors:** Zhiyong Li, Jian Li, Yue Hu, Xinyi Wu, Xiaojuan Duan, Demin Kong, Xiaowen Li, Jin Cheng, Meina Wang

**Affiliations:** 1 Key Laboratory of National Forestry and Grassland Administration for Orchid Conservation and Utilization, The National Orchid Conservation Center of China and the Orchid Conservation & Research Center of Shenzhen, Shenzhen 518114, China; lizhiyong83@hotmail.com (Z.L.); etecology@foxmail.com (J.L.); huyuea0914@126.com (Y.H.); xin-shenhaiyu@163.com (X.W.); comeondxj@126.com (X.D.); damykong@126.com (D.K.); shelwin525@163.com (X.L.); 2 Shenzhen Key Laboratory for Orchid Conservation and Utilization, The National Orchid Conservation Center of China and the Orchid Conservation & Research Center of Shenzhen, Shenzhen 518114, China; 3 Key Laboratory of Chinese Medicinal Resource from Lingnan, Ministry of Education, School of Pharmaceutical Sciences, Guangzhou University of Chinese Medicine, Guangzhou 510006, China; 4 State Key Laboratory of Efficient Production of Forest Resources, Beijing Forestry University, Beijing 100083, China; chengjin@bjfu.edu.cn

**Keywords:** *Dendrobium officinale*, metabolic profiling, tissue specificity, environmental modulation, chemotypic divergence, cultivation optimization

## Abstract

**Highlights:**

**What are the main findings?**

- Metabolomic profiling revealed obvious inter-varietal divergence, and leaves displayed higher metabolic variability compared to stems in *D. officinale*.
- Environmental plasticity: Outdoor cultivation enhanced flavonoid production in D. officinale, while greenhouse cultivation favored alkaloid accumulation in *D. officinale*.

**What are the implications of the main findings?**

- This study elucidates the tripartite interaction between geographical origin, tissue compartmentalization, and environmental cues in shaping chemotypic diversity.
- Our findings establish a framework for optimizing cultivation strategies through targeted environmental adjustments and varietal selection to enhance bioactive compound yields.

**Abstract:**

**Background/Objectives**: *Dendrobium officinale* (*D. officinale*), an endangered ornamental and medicinal orchid, displays significant variability in its bioactive compounds depending on geographical and environmental factors. To decipher these influences, we investigated metabolic divergence across three cultivars (GN, LS, DX) cultivated in greenhouse and outdoor conditions using untargeted metabolomics. **Methods**: Metabolites extracted from stem and leaf tissues were analyzed via UHPLC-Q Exactive Orbitrap MS, and the raw data were processed using XCMS for peak alignment and quantification. Differentially abundant metabolites (DAMs) were identified by multivariate statistical analyses including PCA and OPLS-DA. Metabolic pathways were annotated using KEGG, HMDB, and LIPID Maps databases, with enrichment analysis and visualization performed via TBtools II and Hiplot. **Results**: Metabolite profiling and multivariate analysis revealed distinct chemotypes. The DX cultivar exhibited anthocyanin enrichment in its stems, correlating with a red pigmentation, while GN accumulated specific amino acid derivatives. Tissue-specific metabolic specialization was evident, with leaves displaying greater flavonoid diversity and stems prioritizing lipid and amino acid metabolism. Outdoor cultivation enhanced flavonoid biosynthesis, whereas greenhouse conditions favored alkaloid accumulation. Functional analysis identified both conserved pathways, like phenylpropanoid biosynthesis, and varietal-specific adaptations in amino acid and secondary metabolism. Notably, alkaloid levels declined sharply during plant defoliation. **Conclusions**: Our findings demonstrate that environmental factors and geographical origin synergistically shape the metabolic profiles of *D. officinale*. This provides a scientific basis for optimizing cultivation strategies—through targeted environmental adjustments and varietal selection—to enhance the yield of desired bioactive compounds.

## 1. Introduction

*Dendrobium officinale* (*D. officinale*), an epiphytic orchid native to East Asia, is highly prized for both its ornamental appeal and its extensive use in traditional Chinese medicine (TCM). For centuries, it has been employed to treat a range of metabolic disorders, an efficacy attributed to its rich repertoire of bioactive compounds, including polysaccharides, alkaloids, and flavonoids [1,2]. Modern clinical studies have begun to validate these traditional uses, demonstrating its potential in managing diabetes, gastrointestinal issues, and age-related degenerative conditions [3,4]. This therapeutic potential, combined with its use in nutraceuticals and cosmeceuticals, underscores its significant commercial and medicinal value.

Despite its importance, wild *D. officinale* populations are critically endangered due to overharvesting and habitat loss. While cultivation presents a sustainable alternative, a major challenge has emerged: metabolite variability in farmed plants often compromises their quality equivalence to wild specimens. This discrepancy, driven by differences in environmental factors (e.g., light, water) and genetic diversity [5,6,7,8,9], affects both nutritional quality and therapeutic efficacy. Therefore, a systematic understanding of how these factors shape the metabolome is crucial for developing optimized cultivation strategies that ensure consistent, high-quality yields.

Recent advances in metabolomics now allow for high-resolution mapping of plant chemotypes [10,11]. However, studies on *D. officinale* have been fragmented. There are many varieties of dendrobiums in China; however, only limited varieties are popular, such as *D. officinale* and *D. huoshanense*. Insight into the metabolite profiles of *Dendrobium* species growing under different environmental conditions will provide valuable information for the cultivation and efficient use of distinct *Dendrobium* species. While genomic resources are emerging [12,13,14], comprehensive metabolomic databases are still incomplete, hindering the exploration of germplasm resources and the development of trait-specific breeding programs [15,16,17]. A key knowledge gap remains in understanding how genetic background and environment interact to influence tissue-specific accumulation of bioactive metabolites.

In this study, we integrated untargeted metabolomics with multivariate statistics to unravel the interplay of geographical origin, tissue specificity, and environmental conditions on the metabolome of three *D. officinale* varieties (GN, LS, and DX) originating from distinct Chinese provinces. Our comparative analysis revealed distinct chemical profiles among these cultivars, with inter-varietal differences being more pronounced in leaves than in stems. Kyoto Encyclopedia of Genes and Genomes (KEGG) enrichment analyses delineated these tissue-specific and inter-varietal metabolic divergences. Notably, metabolic divergence correlated with observable phenotypes: DX stems displayed a light red coloration due to anthocyanin accumulation, whereas LS and GN stems remained green. Furthermore, our results demonstrate that environmental conditions (greenhouse vs. outdoor) and growth phases significantly influence natural product accumulation.

Overall, these findings advance our understanding of how geographical and environmental regulation orchestrates metabolic reprogramming in *D. officinale*, providing a roadmap for the targeted cultivation of high-quality medicinal materials.

## 2. Materials and Methods

### 2.1. Reagents and Plant Materials

LC-MS grade methanol, ethanol, and acetonitrile were obtained from CNW Technologies (ANPEL Laboratory Technologies (Shanghai) Inc., Shanghai, China). Formic acid, ammonium acetate and ddH_2_O were purchased from Waters (Milford, MA, USA). Two-year-old *D. officinale* plants, originating from Yunnan (GN), Hunan (LS), and Guangdong (DX) provinces, were cultivated at the National Orchid Conservation Center of China (NOCC), Guangdong Province. Plants were grown under two conditions: a greenhouse (20–30 °C, 70% humidity) and an outdoor bionic environment (natural conditions: 18–32 °C, 13–14 h daylight) on the tree bark. Stem and leaf samples were harvested at both the top-cutting and defoliation stages. Six biological repeats were taken for each sample.

### 2.2. Sample Extraction Procedure

Metabolite extraction was performed as described in our previous study [18] with minor modifications. Stem and leaf tissues were cut into small fragments and ground to a fine powder in liquid nitrogen. For each sample, 100 mg of powder was extracted with 1 mL of pre-chilled extraction solvent (80% methanol, 0.1% formic acid). The mixture was vortexed, sonicated at 40 kHz for 20 min (twice), and then centrifuged at 4 °C for 15 min. The resulting supernatants were filtered through a 0.22 μm syringe tip filter and transferred to LC-MS vials then stored at −80 °C until analysis.

### 2.3. LC-MS/MS Analysis

LC-MS/MS analysis was conducted at Guangzhou Genedenovo Biotechnology Co., Ltd. (Guangzhou, China) using a UHPLC system (1290, Agilent Technologies) (Santa Clara, CA, USA) coupled with a Q Exactive Orbitrap MS from Thermo Fisher Scientific (Waltham, MA, USA). Samples were injected onto a UHPLC HSS T3 column (2.1 mm × 100 mm, 1.8 μm) and eluted with a 12 min linear gradient at a flow rate of 0.5 mL/min. The mobile phase was a mixture of solvent A (0.1% formic acid in water for positive ion mode, or 5 mmol/L ammonium acetate in water for negative ion mode) and solvent B (acetonitrile). The elution gradient was: 0 min, 1% B; 1 min, 1% B; 8 min, 99% B; 10 min, 99% B; 10.1 min, 1% B; and 12 min, 1% B. The QE mass spectrometer was operated in information-dependent acquisition (IDA) mode to acquire MS/MS spectra.

### 2.4. Processing of LC-MS/MS Data

Raw MS data (.raw) were converted to mzML format using ProteoWizard (version 3) and processed with the R package XCMS (version 3.2) for retention time alignment, peak detection, and quantification. Features present in less than 50% of samples within a group (including QC) were filtered out. Data were normalized to an internal standard [19], and missing values were imputed with half the minimum value found in the dataset [20]. The resulting data matrix contained retention time (RT), mass-to-charge ratio (*m*/*z*), and peak intensity. Peaks were annotated using OSI-SMMS (version 1.0, Dalian Chem Data Solution Information Technology Co. Ltd.) by matching against an in-house MS/MS database.

### 2.5. Multivariate Statistical Analysis

For an initial overview of sample grouping, principal component analysis (PCA) was applied to all samples using R (http://www.r-project.org/(accessed on 12 January 2025)). To maximize group discrimination and identify key variables, orthogonal projection to latent structures-discriminant analysis (OPLS-DA) was performed using SIMCA 14.1. Model validity was assessed using R^2^ and Q^2^ goodness-of-fit parameters, along with cross-validation and a 200-permutation test [21].

### 2.6. Differentially Abundant Metabolites (DAMs) Analysis

Differentially abundant metabolites (DAMs) between two groups were identified using a combination of a variable importance in projection (VIP) score ≥ 1 from the OPLS-DA model, a *p*-value < 0.05 from a *t*-test, and an absolute log2 fold change (|log2(FC)|) ≥ 1. Metabolites were annotated using the KEGG (https://www.genome.jp/kegg/pathway.html) (accessed on 20 January 2025), HMDB (https://hmdb.ca/metabolites) (accessed on 20 January 2025), and LIPIDMaps (http://www.lipidmaps.org/) (accessed on 20 January 2025) databases.

### 2.7. Data Analysis and Visualization

For clustering heatmaps, data were normalized to the average signal intensity of DAMs and visualized using TBtools II [22]. Metabolites were mapped to KEGG pathways [23] for enrichment analysis, with results displayed as bubble charts generated on Hiplot (https://hiplot.cn) (accessed on 13 February 2025).

## 3. Results

### 3.1. Metabolomic Profiling Reveals Distinct Chemotypes in Three D. officinale Varieties

**Figure 1:**
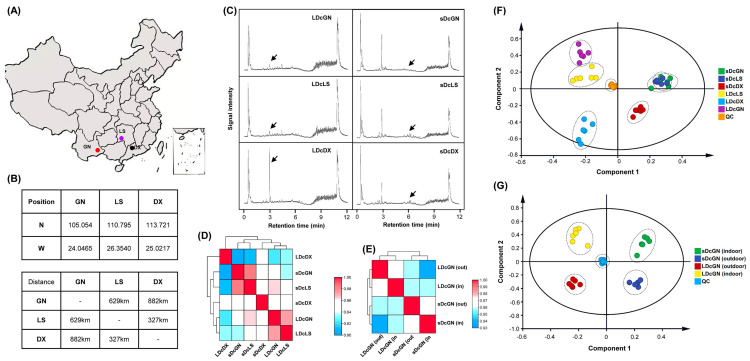
Geographical and multivariate chemical analysis of *D. officinale* varieties. (**A**) Geographical map of China highlighting source areas of three *D. officinale* varieties with positional markers. (**B**) Tabulated coordinates (latitude N, longitude W) and inter-location distances (km). (**C**) Chromatograms of six groups depicting signal intensity versus retention time (min) with key peaks arrowed. (**D**,**E**) Correlation heatmaps demonstrating variable distributions across groups (red = high, blue = low). (**F**,**G**) Location-tissue groups identified by OPLS-DA analysis (locations: Guangnan from Yunan province—GN, Langshan from Hunan province—LS, Danxia from Guangdong province-DX; L: leaf; s: stem). QC and environmental markers (indoor/outdoor) included. QC: quality control.

This study investigated three groups of *D. officinale* cultivated in greenhouse and outdoor bionic environments. The original germplasms were sourced from Guangnan County (GN), Yunnan Province; Langshan (LS), Hunan Province; and Danxia Mountain (DX), Guangdong Province (Figure 1A). These locations exhibit minimal longitudinal and latitudinal differences, with DX located 327 km from LS and 682 km from GN (Figure 1B). Preliminary UHPLC analysis revealed distinct ion flow signal intensities in leaf extracts, particularly for the DX variety near the 3- and 4.5 min retention times, suggesting a unique chemical profile (Figure 1C). Stem ion flow signals were consistent across all varieties except for reduced intensity in the GN variety at the 2.5- and 6 min retention time.

Based on untargeted metabolomic profiling, 3459 and 1428 small molecule metabolites were identified using positive and negative ion modes, respectively. A correlation heatmap of all identified metabolites demonstrated that the DX variety possessed the most distinct metabolic profile, showing clear separation from the more closely related LS and GN varieties (Figure 1D). OPLS-DA score plots further highlighted tissue-specific metabolic specialization, with inter-varietal differences being more pronounced in leaves than in stems (Figure 1F). The influence of the cultivation environment was also evident, as a comparison of indoor- and outdoor-grown GN plants revealed significant shifts in metabolite accumulation patterns (Figure 1G).

### 3.2. Tissue-Specific and Variety-Specific Metabolic Variation

**Figure 2:**
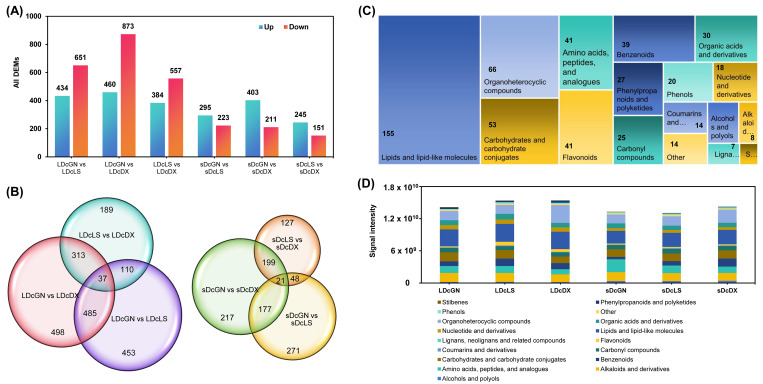
Integrated analysis of DAMs and metabolomic features in *D. officinale* varieties. (**A**) Bar charts showing counts of upregulated/downregulated DAMs across pairwise comparisons. (**B**) Venn diagram depicting DAM overlaps among three comparison groups. (**C**) Classified metabolite overview with total counts for each class. (**D**) Heatmap comparing metabolite intensities across six experimental groups. Abbreviations: Guangnan (GN), Langshan (LS), Danxia (DX).

To systematically characterize these differences, we identified DAMs using stringent criteria (VIP ≥ 1, |log2(FC)| ≥ 1, *p* < 0.05). Consistent with the OPLS-DA results, leaves exhibited far greater metabolic variability between varieties than stems. For example, the comparison between GN and LS leaves yielded 1085 DAMs, whereas the same comparison in stems produced only 518 (Figure 2A). Furthermore, Venn diagram analysis showed minimal overlap in the sets of DAMs from different varietal comparisons, indicating that each variety possesses a unique metabolic fingerprint (Figure 2B).

The 576 unique DAMs that could be annotated were classified into 11 major categories, with lipids, organoheterocyclic compounds, and carbohydrates being the most abundant (Figure 2C). Notably, this included 41 flavonoids and 8 dendrobine alkaloids. Quantitative analysis revealed clear tissue-specific accumulation patterns. For instance, GN stems were enriched in amino acid derivatives, while LS and DX leaves accumulated higher levels of flavonoids (Figure 2D). This data provides a detailed map of the striking inter-varietal and tissue-specific metabolic divergence in *D. officinale*.

### 3.3. Functional Enrichment Analysis Reveals Conserved and Specialized Metabolic Pathways

**Figure 3:**
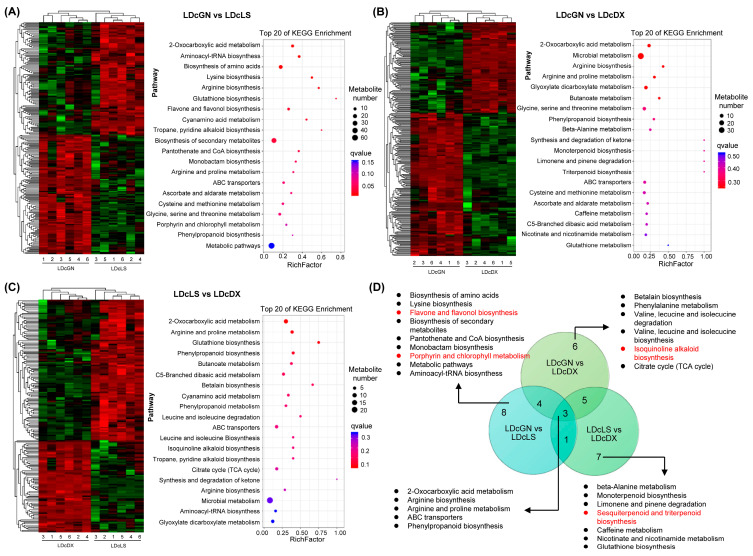
KEGG enrichment analysis of leaves-derived DAMs from *D. officinale* varieties. (**A**–**C**) Heatmaps showing DAM relative intensities (red = high, green = low) with top 20 enriched pathways ranked via bubble charts (blue = high, red = low enrichment scores). (**D**) Venn diagram illustrating shared/distinct KEGG pathways across three variety comparisons. Abbreviations: Guangnan (GN), Langshan (LS), Danxia (DX).

**Figure 4:**
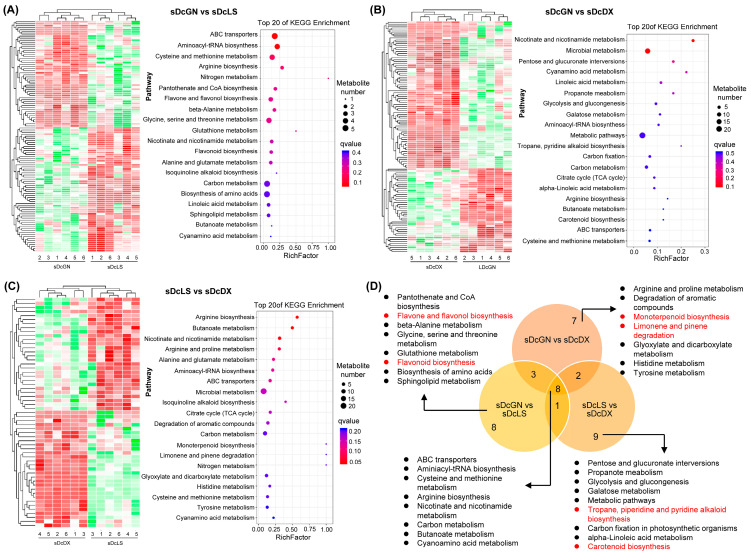
KEGG enrichment analysis of stems-derived DAMs from *D. officinale* varieties. (**A**–**C**) Heatmaps showing DAM relative intensities (red = high, green = low) with top 20 enriched pathways ranked via bubble charts (blue = high, red = low enrichment scores). (**D**) Venn diagram illustrating shared/distinct KEGG pathways across three variety comparisons. Abbreviations: Guangnan (GN), Langshan (LS), Danxia (DX).

To understand the biological processes underlying these metabolic differences, we performed KEGG enrichment analysis on the DAMs from leaves (Figure 3) and stems (Figure 4). In leaves, the comparison between GN and LS highlighted significant enrichment in amino acid and flavonoid metabolism (Figure 3A). The GN vs. DX comparison revealed additional differences in terpenoid and microbial metabolism pathways (Figure 3B), while LS vs. DX pointed to variations in alkaloid biosynthesis (Figure 3C). Despite these varietal specializations, a core set of pathways, including arginine biosynthesis and phenylpropanoid biosynthesis, were enriched in all three comparisons, suggesting they are fundamental to *D. officinale* metabolism (Figure 3D).

Similar analysis of stem DAMs revealed shared pathways across varieties, such as carbon and amino acid metabolism, but also highlighted tissue-specific differences. For instance, GN displayed unique flavonoid metabolism in stems compared to LS, whereas LS exhibited distinct alkaloid and carotenoid pathways relative to DX (Figure 4D). These results delineate a metabolic architecture in *D. officinale* consisting of both conserved core pathways and specialized, variety-specific branches.

### 3.4. PCA Confirms Class-Specific Metabolic Differentiation Among Varieties

**Figure 5:**
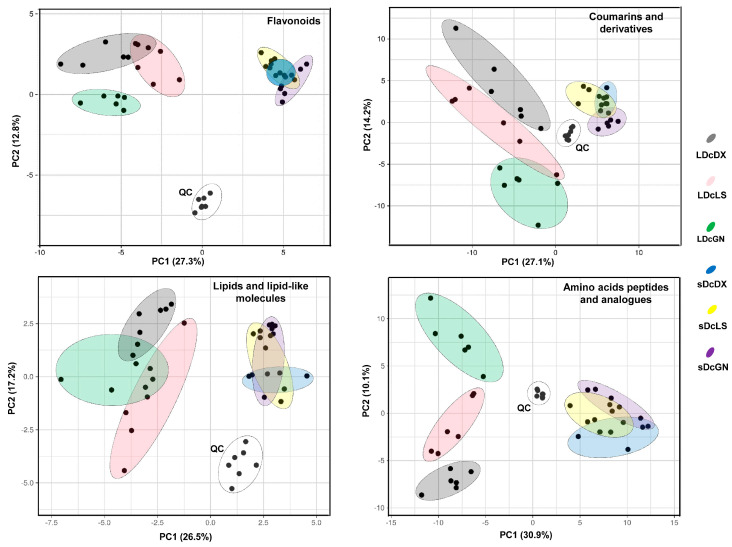
PCA score plot of category-specific metabolic differentiation in *D. officinale* varieties. Analyses include leaf (L) and stem (s) tissues from three varieties (GN, LS, DX) alongside quality control (QC) samples. QC samples (gray) validate analytical consistency. Abbreviations: Guangnan (GN), Langshan (LS), Danxia (DX).

A more detailed PCA within four key metabolite classes—flavonoids, coumarins, lipids, and amino acids—confirmed the distinct chemotypes of the three varieties (Figure 5). Consistent with our previous findings, leaves were the primary site of metabolic differentiation. For instance, GN displayed a distinct flavonoid distribution cluster, separated from both DX and LS (Figure 5, “Flavonoids” panel). No overlap in flavonoid profiles was observed between GN and the other two varieties, suggesting unique flavonoid biosynthetic pathways in GN. In addition, GN shared lipid distributions with both DX and LS, whereas DX and LS exhibited completely non-overlapping lipid profiles (Figure 5, “Lipids and lipid-like molecules” panel). Complete separation of all three varieties was observed in the amino acid and peptide profiles, further underscoring the genetic basis for their metabolic uniqueness.

### 3.5. Flavonoid Profiling Reveals Anthocyanin-Driven Phenotypic Divergence in DX Stems

**Figure 6:**
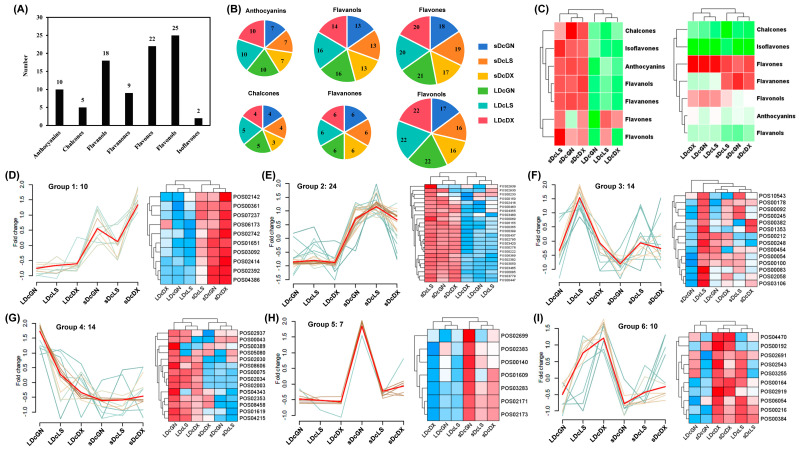
Comprehensive flavonoid profiling in *D. officinale* varieties. (**A**) Quantitative analysis and (**B**) distribution patterns of seven flavonoid classes in stems/leaves across three varieties. (**C**) Heatmap showing total flavonoid content (red = high, green = low). (**D**–**I**) Hierarchical clustering of six multivariate groups with flavonoid-specific signal intensities in heatmaps (red = high, blue = low), total flavonoid counts labeled per group. Abbreviations: Guangnan (GN), Langshan (LS), Danxia (DX).

Further investigation of flavonoid characteristics in three *D. officinale* varieties revealed the identification and annotation of 91 flavonoids, including 10 anthocyanins, 5 chalcones, 18 flavanols, 9 flavanones, 22 flavones, 25 flavonols, and 2 isoflavones (Figure 6A, Table S2). While the diversity of flavonoid subclasses was similar across tissues, leaves exhibited a slightly higher number of flavanols, flavonols, and anthocyanins (Figure 6B). Hierarchical clustering of flavonoids based on their ion intensity revealed significantly higher flavonoids content in stems than leaves across all varieties, with exceptionally high flavone levels in LS and DX leaves (Figure 6C).

The 91 flavonoids were further categorized into six distinct groups based on their distribution patterns. Groups 1 and 2 contained 34 flavonoids with elevated levels in stems across all three varieties (Figure 6D,E). Group 3 highlighted 14 flavonoids with high abundance in LS leaves (Figure 6F). Groups 4 and 5 represented 14 and 7 flavonoids specifically abundant in GN leaves and stems, respectively (Figure 6G,H). Group 6 identified 10 flavonoids with elevated levels in LS and DX leaves (Figure 6I). These clustering patterns underscore the 304 tissue-specific and chemotype-dependent regulation of flavonoid biosynthesis in *D. officinale* varieties.

**Figure 7:**
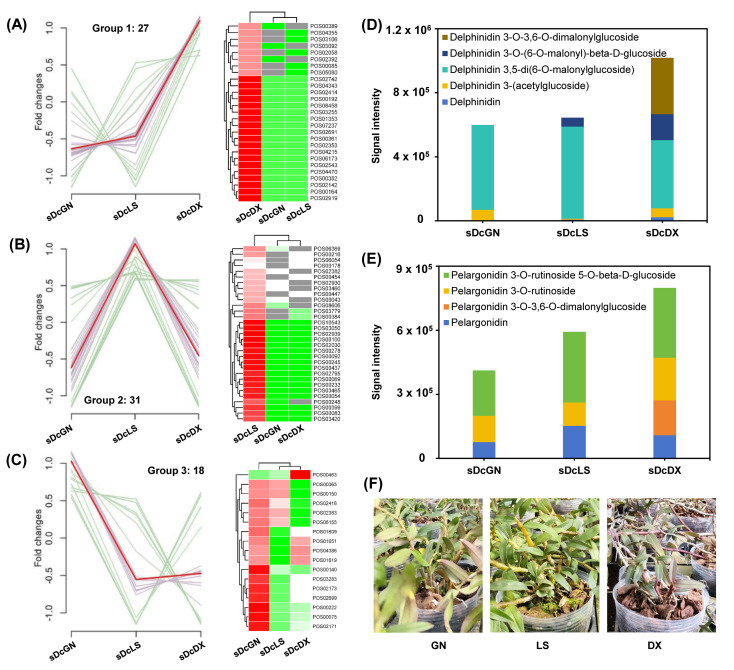
Anthocyanin-driven metabolic divergence and phenotypic correlation in *D. officinale* stems. (**A**–**C**) Hierarchical clustering of stem-derived flavonoids from three *D. officinale* varieties and heatmaps showing relative signal intensities of flavonoids in each group (red = high, green = low). Total number of flavonoid for each group as indicated. (**D**,**E**) Comparative quantification of delphinidin/pelargonidin and their derivatives in stem tissues. (**F**) Greenhouse-grown phenotypic comparison among three *D. officinale* varieties. Abbreviations: Guangnan (GN), Langshan (LS), Danxia (DX).

A focused analysis of stem flavonoids revealed three distinct varietal clusters (Figure 7A–C). Group 1, containing 27 high-abundance flavonoids, was predominantly observed in the DX variety. Groups 2 and 3 exhibited 31 and 18 high-abundance flavonoids in the LS and GN varieties, respectively. Detailed analysis of DX-specific flavonoids identified anthocyanin as the predominant subclass, with liquid chromatography-ion flow signal statistics confirming elevated levels of delphinidin and pelargonidin derivatives (Figure 7D,E). This metabolic divergence correlated perfectly with a visible phenotype: the stems of the DX variety displayed a light red coloration, whereas the LS and GN stems remained green (Figure 7F). This finding directly links a specific chemotypic trait (anthocyanin accumulation) to a morphological characteristic (stem color).

### 3.6. Environmental Conditions and Growth Stage Dynamically Modulate Secondary Metabolism

**Figure 8:**
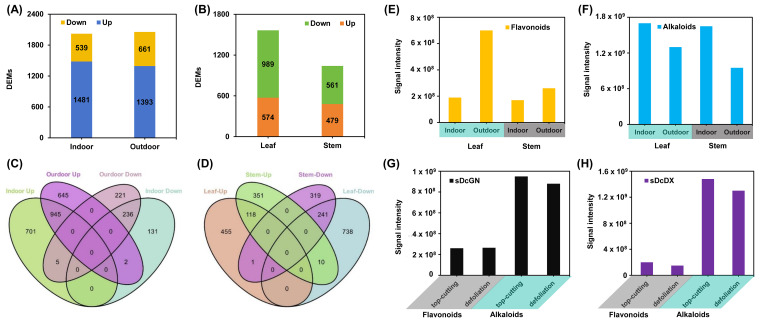
Environmental and developmental regulation of metabolite dynamics in *D. officinale*. (**A**,**B**) Bar chart showing DAMs between stems/leaves and indoor/outdoor conditions. (**C,D**) Venn diagram illustrating DAM overlaps from (**A**,**B**) comparisons. (**E**,**F**) Comparison of flavonoids (**E**) and alkaloids (**F**) Flavonoid and alkaloid quantification in DX variety under indoor/outdoor cultivation. (**G**,**H**) Comparative analysis of flavonoids and alkaloids in GN/DX varieties during top-cutting and defoliation stages. Abbreviations: Guangnan (GN), Danxia (DX).

Using the GN variety as a model, we next investigated the impact of cultivation environment (greenhouse vs. outdoor) on the metabolome. While the total number of detected metabolites was similar between conditions, a large set of compounds showed environment-dependent accumulation (Figure 8A–D). Notably, outdoor cultivation significantly enhanced flavonoid biosynthesis in both stems and leaves, whereas indoor, greenhouse conditions favored the accumulation of alkaloids (Figure 8E,F).

Furthermore, the growth phase exerted a strong influence on metabolite levels. While flavonoid content in GN remained relatively stable between the top-cutting and defoliation stages, alkaloid levels dropped sharply during defoliation (Figure 8G). A similar decline in both flavonoid and alkaloid content during defoliation was observed in the DX variety (Figure 8H). These results demonstrate that secondary metabolism in *D. officinale* is highly plastic, dynamically regulated by both environmental cues and developmental programs.

### 3.7. Comparative Analysis of Climate Change Across Three Distinct Geographical Origins of D. officinale

**Figure 9:**
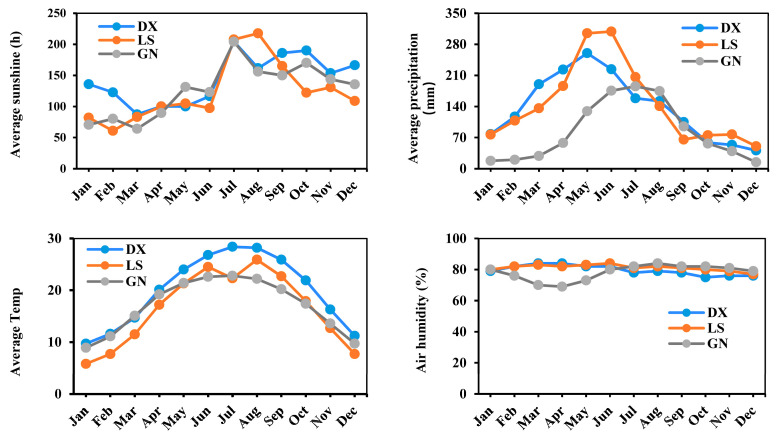
Comparative study of the climate data from three geographical origins of *D. officinale*. Abbreviations: Guangnan (GN), Langshan (LS), Danxia (DX).

The regional climatic variations may contribute to the geographical differentiation observed in *D. officinale* populations across the three areas. Hence, we turned to investigate the climate change from the geographical origins of *D. officinale*. Based on climate data from the China Meteorological Administration and the National Bureau of Statistics over the past five years, the climatic characteristics of three regions—DX, LS, and GN—were investigated. The results revealed distinct regional climate patterns that may influence the genetic variation in *D. officinale* (Figure 9). In terms of sunshine duration, the DX region experienced relatively longer average sunlight hours during spring and winter, whereas the LS region received more sunlight in autumn. Concerning precipitation, both DX and LS exhibited higher average rainfall levels, with LS receiving particularly abundant rainfall during the summer months. The average monthly temperature was highest in the DX region. In contrast, differences in air humidity among the three regions were minimal.

## 4. Discussion

This comprehensive metabolomic investigation of three *D. officinale* varieties provides critical insights into how geographical, environmental, and tissue-specific factors interact to shape natural product biosynthesis. Our findings reveal profound chemotypic diversity among varieties, a strong tissue-specific allocation of metabolites, and a dynamic metabolic plasticity in response to cultivation conditions. These results not only advance our fundamental understanding of *Dendrobium* metabolism but also establish a practical framework for optimizing cultivation strategies to enhance the production of bioactive compounds.

### 4.1. Geographic Origin Determine Chemotypic Identity

Environmental factors of different origins may have a great influence on *D. officinale* metabolites. Previous study presented that more flavonoids were accumulated in the RS (Shaoguan from Guangdong Province) samples, although the samples from all three sources (two other varieties from Guangxi and Zhejiang Province) contained similar metabolites [24]. Another report showed that the polysaccharide content is highly variable from four different origins: Anhui province (AH), Guangxi province (GX), Guizhou province (GZ) and Yunnan province (YN) [25]. These findings imply that factors such as geographical distribution play an important role in the chemical composition and bioactivity of *D. officinale*.

In our study, the clear metabolic separation of the DX variety from LS and GN, particularly its unique anthocyanin-rich profile, suggests that localized geographical adaptation to specific habitats drives chemotypic divergence. This divergence likely stems from adaptive responses to environmental stressors—such as microclimatic conditions—that modulate the differential expression of key biosynthetic pathways. For instance, DX stems accumulated anthocyanins (e.g., delphinidin and pelargonidin derivatives), which directly correlated with their light red pigmentation, a phenotypic trait conspicuously absent in the green-stemmed LS and GN varieties (Figure 7D–F).

The distinct climatic characteristics of the DX region, notably its longer sunshine duration in spring and winter and its highest average monthly temperatures (Figure 9), provide a plausible environmental link to this observed deep-red stem phenotype. The combination of prolonged light exposure and elevated temperatures may act as an abiotic stressor, triggering enhanced photoprotective mechanisms. Anthocyanins, accumulated in DX stems, are known to mitigate photo-oxidative damage caused by high light intensity and temperature stress [26]. Therefore, the red pigmentation may represent an adaptive response to the local microclimate, where increased anthocyanin biosynthesis helps reduce oxidative stress and optimize photosynthetic performance. This environmentally induced chemotypic divergence underscores how fine-tuned regulation of flavonoid pathways facilitates plant adaptation to ecological niches.

### 4.2. Core and Specialized Pathways Define the Metabolic Landscape

KEGG enrichment analysis revealed both conserved and divergent metabolic pathways among varieties (Figure 3 and Figure 4). Arginine biosynthesis and phenylpropanoid pathways were universally enriched across all comparisons, underscoring their central roles in nitrogen metabolism and as gateways to diverse secondary metabolite synthesis (Figure 3D and Figure 4D). These universally enriched pathways likely represent the core metabolic backbone essential for fundamental physiological processes in *D. officinale*.

However, varietal contrasts unveiled unique pathway specializations that define each variety’s metabolic identity. The GN vs. LS comparison highlighted flavonol synthesis and porphyrin metabolism, while GN vs. DX featured TCA cycle activation and isoquinoline alkaloid biosynthesis (Figure 3 and Figure 4). Such divergence implies that varieties prioritize distinct metabolic strategies under geographical or environmental pressures. For example, GN’s apparent investment in the TCA cycle may enhance carbon flux toward amino acid derivatives, which aligns with its observed stem-specific amino acid accumulation. Conversely, LS’s enrichment in flavonol synthesis pathways corresponds with its higher leaf flavonoid content. These findings mirror reports in crop plants, where pathway specialization underpins stress tolerance and nutritional quality [27,28,29,30]. The coexistence of both core and specialized pathways within the same species highlights the metabolic flexibility that allows *D. officinale* to maintain essential functions while adapting to local environmental conditions.

### 4.3. Tissue Specificity as a Strategy for Ecological and Metabolic Specialization

The consistent observation of greater metabolic divergence in leaves compared to stems underscores their distinct ecological and physiological roles. As the primary sites of photosynthesis and environmental interaction, leaves likely prioritize a diverse and inducible arsenal of defense-related metabolites, such as flavonoids and alkaloids, to adjust biotic and abiotic stresses [31]. Indeed, dynamic phytochemical profiles to bioactivity in leaves have been proved with maximum anti-inflammatory and antioxidant activity in July by vanillic acid 4-β-D-glucoside, schaftoside, and rutin as key bioactive contributors [32]. Stems, on the other hand, may focus on a more stable set of structural and storage compounds. The accumulation of different flavonoid classes in leaves (flavonols) versus stems (flavanones) further supports this tissue-level metabolic partitioning and suggests that different plant parts could be targeted for different bioactive compounds. The flowers and leaves are also found particularly enriched in bioactive flavonoids and alkaloids, suggesting remarkable metabolic diversity and functional potential of *D. officinale* [33].

Among all metabolite classes, flavonoids emerged as key drivers of both inter-varietal and tissue-specific differences. Although stems generally harbored higher total flavonoid content, specific subclasses showed distinct distribution patterns: flavones were particularly abundant in LS and DX leaves, while flavonols dominated in leaf tissues across all varieties (Figure 6C). Hierarchical cluster analysis revealed six distinct flavonoid distribution groups, including GN-specific leaf clusters (Group 4) and DX/LS-shared leaf clusters (Group 6), indicating complex regulatory mechanisms governing flavonoid biosynthesis.

The DX variety’s anthocyanin-rich profile, linked to stem pigmentation, suggests preferential activation of the phenylpropanoid pathway under specific environmental cues. These findings resonate with reports on other *Dendrobium* species, where flavonoid diversity correlates with both geographical lineage and ecological adaptation [34,35]. Consistent with this, a previous study demonstrated that flavonoids in *D. officinale* from three different Chinese locations (Zhejiang province, Guangxi province and Guangdong province in China) differed both quantitatively and qualitatively [36], further supporting the notion that geographic origin shapes flavonoid metabolism.

### 4.4. Environmental Plasticity Offers a Roadmap for Optimized Cultivation

The dramatic shifts in metabolite profiles in response to environment and growth phase demonstrate the profound metabolic plasticity of *D. officinale*. For instance, the polysaccharide content of the *D. officinale* significantly varies under tree epiphytic cultivation, stone epiphytic cultivation, and greenhouse cultivation [37]. A study with multiple cultivation modes (greenhouse, GC; understory, UC; and simulated wild, SWC) across China for *D. officinale* provides critical insights into the influence of cultivation modes on the phytochemical profiles and bioactivities of *D. officinale* [38]. For example, understory cultivation (UC) yielded products with superior total phenolic/flavonoid content compared to the greenhouse (GC) and simulated wild (SWC) modes.

In our case, the outdoor promotion of flavonoids, likely a response to higher light stress, and the indoor promotion of alkaloids, possibly linked to more controlled nutrient conditions, provide a clear rationale for environment-specific cultivation (Figure 8E,F). These observations align with studies highlighting environmental plasticity in secondary metabolism, where light intensity, temperature, and nutrient regimes act as pivotal regulators [39,40,41]. Importantly, the sharp decline in alkaloid content during defoliation (Figure 8G,H) underscores the critical importance of harvest timing, suggesting that the top-cutting stage is optimal for alkaloid yield.

This knowledge can be directly translated into tailored cultivation protocols: outdoor systems for flavonoid-rich harvests and greenhouse conditions for alkaloid-focused production. Such environment-specific approaches could enable producers to manipulate metabolic outcomes predictably, maximizing the accumulation of desired bioactive compounds. Furthermore, understanding the geographical basis of varietal differences in environmental responsiveness could facilitate the selection or breeding of cultivars optimized for specific cultivation systems or target metabolites.

### 4.5. Limitations and Future Directions

While this study provides a robust metabolomic framework, several limitations warrant consideration. First, the untargeted approach, though comprehensive, may overlook low-abundance metabolites with potential bioactivity. Future work should incorporate targeted quantification of key bioactive compounds to refine functional interpretations. Second, the observational nature of our environmental correlations limits causal inference; controlled experiments manipulating specific environmental variables (e.g., light intensity, photoperiod, temperature) would strengthen our understanding of causal relationships. Third, integrating transcriptomic or proteomic data with our metabolomic profiles would help uncover the regulatory genes and enzymes governing the observed metabolic patterns. Such multi-omics approaches could identify molecular markers for trait-specific breeding and reveal conserved regulatory networks underlying metabolic plasticity. Finally, expanding the geographical sampling to include additional populations and cultivation sites would enhance the generalizability of our findings and support the development of comprehensive metabolomic databases for *Dendrobium* germplasm resources.

## 5. Conclusions

This study deciphers the complex interplay of geographical, environmental, and tissue-specific factors that govern the metabolite biosynthesis in *D. officinale*. Our findings highlight the chemotypic uniqueness of geographically distinct varieties, the ecological rationale behind tissue-specific metabolite allocation, and the significant malleability of metabolic pathways under environmental and developmental cues. These insights offer a scientific basis for the geo-authentic cultivation and pharmacological utilization of *D. officinale*, providing practical strategies for producing high-value metabolites through optimized environmental management and informed geographical selection.

## Data Availability

The original contributions presented in this study are included in the article and supplementary material. Further inquiries can be directed to the corresponding author.

## Supplementary Materials

The following supporting information can be downloaded at: https://www.mdpi.com/article/10.3390/metabo16040279/s1, Table S1: Annotated differential abundant metabolites from stems and leaves of *D. officinale*; Table S2: Identified flavonoids from stems and leaves of *D. officinale*.
